# Prevention of re-establishment of malaria

**DOI:** 10.1186/s12936-021-03781-4

**Published:** 2021-05-31

**Authors:** Allan Schapira, Anatoly Kondrashin

**Affiliations:** 1 Bicol University College of Medicine, Legazpi City, Philippines; 2 Martsinovski Institute of Medical Parasitology, Tropical and Vector-Borne Diseases, Sechenov University, 119 435 Moscow, Russia

**Keywords:** Malaria, Elimination, Prevention of re-establishment, Surveillance, Vigilance, Mortality, Travellers’ health

## Abstract

The current consensus on prevention of re-establishment of malaria is based on the following principles: (1) Fundamental role of general health services; (2) Surveillance; (3) Vector control; (4) Border actions; (5) Intersectoral collaboration. These principles are critically reviewed, and it is pointed out that alertness of the general health services to suspected malaria (*vigilance*) needs to be maintained everywhere, while health education is rational only if targeting high-risk sub-populations. It is argued that prevention of re-establishment of malaria transmission should be integrated with prevention of malaria mortality in cases of imported malaria, and that this requires collaboration with entities dealing with travellers’ health and the availability of chemoprophylaxis and other measures for travellers to malaria endemic countries.

## Background and findings

Since the first global malaria eradication campaign, the consensus on prevention of re-establishment of malaria (POR) has changed little. In the following, the principles of POR as identified in a recently published review [1], and in World Health Organization (WHO) guidance documents [2, 3] are summarized together with some suggestions about strengthening of current practice. The views expressed by the authors are largely based on their experiences in WHO missions for certification of malaria-free status and in malaria programme reviews in countries approaching elimination.

## Fundamental role of general health services

The basis for POR, as for the achievement of malaria-free status, is the early detection, management, and reporting of any malaria case, whatever its origin, by the health services. For early detection to be possible, health services must be universally distributed, and accessible, and primary level service providers must be alert to the possibility of malaria in patients with symptoms compatible with a locally appropriate case definition of suspected malaria. All programmes in the POR phase include training of health care providers and supplies of diagnostics and medicines, but the training does not always reach the primary level, and, consequently, case detection is delayed. In Sri Lanka, the absence of indigenous malaria led to a loss of awareness among the medical profession, resulting in delayed diagnosis despite the widespread availability of malaria diagnosis service, and this had to be remedied systematically by a multi-pronged educational approach [4]. The readiness of the general health services to deal with suspected malaria should be monitored. In the past, annual blood examination rate (ABER) was considered a good indicator; the validity may be compromised, because the numerator can be inflated by misguided active case detection. Annual blood examination rate based on data exclusively from passive case detection could be better, but it is difficult to establish benchmarks, because the occurrence of patients with a relevant travel history varies in time and space. The “1-3-7”approach, which has proven practical and useful, monitors the timeliness of reporting of detected cases [5], but ignores cases that are missed or not reported. Some scientists are exploring questionnaire surveys and simulated patient methodologies [6]. Such methods are more laborious, but also more valid.

## Surveillance

A malaria programme, a vector-borne disease control programme or an epidemiological service must regularly assess the geographical extent of receptivity (risk of mosquito transmission) and vulnerability (malaria importation risk). This central unit must ensure proper classification, reporting and recording for all malaria cases and the identification and management of foci. It is also responsible for maintaining the alertness of general health services, the quality and availability of confirmatory diagnosis, updated treatment policy and availability of anti-malarials. Finally, it should be able to mount active case detection in response to cases in receptive areas or heightened risk; a recent investigation in Sri Lanka found that reactive case detection, particularly among travel cohorts, is very important [7].

According to some WHO texts, **surveillance** is replaced by **vigilance** in a country, where malaria has been eliminated [8]. This shift in terminology is not applied to any other disease under elimination. The WHO framework for malaria elimination published in 2017 and most recent research articles rightly use the term surveillance also for the POR phase. However, the authors believe that the term vigilance is usefully applied to the alertness of the general health services, which should be one fundamental component of malaria surveillance aiming to prevent re-establishment.

## Vector control

Vector control services supported by entomological monitoring and investigation need to be maintained, to allow rapid intervention if there is an outbreak (**active focus**) or an imported case occurs in a receptive area [9]. While the strategic guidance is unchanged, it is regrettable that the term ‘**potential focus**’ used in earlier texts for the latter situation [2], is no longer promoted [3], although it is practical for planning (how many potential foci of what size are expected in an area per year?), action (delimitation of the area to receive intervention based on local realities) and monitoring (what level of coverage was achieved on time in those foci?). Additionally, proactive vector control may be warranted in some countries, in areas where high receptivity and high importation risk overlap.

## Border actions

In some situations, POR programmes include screening of people arriving from endemic countries. This may be costly [10] and is not universally recommended [3]. The parasite density levels in asymptomatic carriers are often not detectable by microscopy or RDTs, and, there is no method for detecting hypnozoites. Possibly, advice on seeking health care would be more valuable, but the two approaches are not mutually exclusive; they could even be synergistic. Primaquine or tafenoquine treatment of incoming glucose-6-phosphate dehydrogenase (G6PD) normal persons at high risk of carrying hypnozoites would theoretically be effective, but has not been practiced on a large scale and appears rather aggressive. In some border areas with a high risk of importation, cross-border collaboration should be considered. To be effective, it must be designed according to the local situation and the interests of the countries involved.

## Intersectoral collaboration

The risk of re-establishment of transmission is often linked to persons arriving to seek work, though it may also be refugees, returning travellers and many other groups [11]. Generally, prevention and case management for such persons require collaboration with employers or agencies looking after refugees or other migrants. There are many options for inter-sectoral collaboration, which can be adopted according to local risks.

## Health education

It is sometimes stated that the populations in malaria eliminating countries should be kept educated about the symptoms of malaria, so that they will seek medical care and be diagnosed early. However, it seems odd to tell the population of a country that malaria has been eliminated, and then, that they should still be thinking about malaria, if they have fever. For a person with an unexplained fever, the possibility of serious disease should be the motivation for seeking care-seeking; thinking about malaria should be the responsibility of the health care provider [12]. Still, it is rational to educate persons departing and arriving from endemic countries, and those living in receptive border areas with extensive population movement [13].

One weakness of POR strategies as practiced by some countries which have recently eliminated malaria is the lack of linkage with **Travellers’ Health** and the imperative to prevent malaria deaths. Chemoprophylaxis for travellers should reduce the risk of importation of malaria cases. Yet, the effect in relation to risk of re-establishment of transmission may be limited, because the traveller returning to a malaria-free country may be less likely than a job-seeker from an endemic country to stay in a receptive area under conditions conducive to transmission [14]. For travellers to countries, which are endemic for vivax malaria, there is again the problem of hypnozoites, which are not killed by most of the medicines used for chemoprophylaxis. For people without G6PD deficiency, primaquine or tafenoquine could be an option for chemoprophylaxis [15]. In most of the countries which have recently eliminated malaria or are on track towards that objective, Travellers’ Health services are neglected, with negative consequences, not only concerning malaria. Yellow fever vaccination clinics may be a starting point for building such services. Some malaria programmes could undertake operational research to identify which travellers are at high risk of contracting malaria abroad and work out tailored strategies. Often, they are jobseekers, meaning that labour recruitment agencies should be prioritized. Strategies must go beyond education to ensure availability and affordability of appropriate medicines and personal protection products.

In 2019, in a malaria programme review in a country close to elimination, but with one remaining problem-area, the review team observed that the case fatality rate of falciparum malaria was higher among imported cases than in the remaining endemic area in the country. From an equity and elimination viewpoint, the programme had its priorities right, but more could be done to ensure prevention and effective management of imported cases. It does not appear reasonable for a Ministry of Health to triumphantly declare that the country is malaria-free if people continue to succumb to this disease after contracting it abroad. Targeting all efforts exclusively on receptive areas is also problematic because receptivity is both relative and dynamic. Integrating prevention of re-establishment of transmission with prevention of mortality is likely to be beneficial for government commitment and maintenance of the interest of the medical profession.

## Conclusions

A malaria surveillance system is needed both before and after interruption of transmission. One essential component of this system in areas and countries, where malaria has been eliminated is the vigilance of the general health services, starting with the ability of primary level providers to identify and deal with (by referral, or diagnosis, treatment, and reporting) cases of suspected malaria. That vigilance must be ensured and monitored by the central unit responsible for malaria surveillance. In addition to surveillance, POR strategies must include vector control, border actions, intersectoral collaboration and health education. POR should prioritize risks in receptive areas. However, POR strategies are likely to have broader support if they are integrated with efforts to prevent mortality in cases of imported malaria. This requires close collaboration with travellers’ Health (in some countries even the development of such services) and attention to the availability of chemoprophylaxis and other measures for travellers to endemic countries.

## Data Availability

Not applicable.

## Abbreviations

G6PD: Glucose-6-phosphate dehydrogenase
POR: Prevention of re-establishment of malaria transmission
